# COVID-19 in hemodialysis patients: New insights into metabolomic profile dynamics from 60 days pre- to 60 days post-diagnosis

**DOI:** 10.1371/journal.pone.0346687

**Published:** 2026-04-17

**Authors:** Gabriela F. Dias, Chenxi Fan, Maggie Han, Xiaoling Wang, Ohnmar Thwin, Lemuel Fuentes, Xin Wang, Hanjie Zhang, Wensheng Guo, Peter Kotanko, Nadja Grobe, Yuedong Wang

**Affiliations:** 1 Renal Research Institute, New York, New York, United States of America; 2 Department of Statistics and Applied Probability, University of California, Santa Barbara, California, United States of America; 3 Department of Biostatistics and Epidemiology, Perelman School of Medicine, University of Pennsylvania, Philadelphia, Pennsylvania, United States of America; 4 Icahn School of Medicine at Mount Sinai, New York, New York, United States of America; University of California Riverside, UNITED STATES OF AMERICA

## Abstract

**Background:**

Maintenance hemodialysis patients experience higher morbidity and mortality from COVID-19, partly due to comorbidities like diabetes and cardiovascular disease. However, kidney disease-related metabolic processes may also contribute.

**Methods:**

In this prospective, multi-center, observational study, we analyzed 201 routine serum samples from 30 hemodialysis patients (average age 59.2 ± 13.3 years, 57% male) with confirmed COVID-19, collected from 60 days before and 60 days after diagnosis. Untargeted liquid chromatography/mass spectrometry was used to profile metabolites. Linear and semi-parametric mixed-effects models were applied to assess changes across four phases: baseline (−60 to −15 days), putative incubation period (PIP; −14–0 days), acute (1–14 days), and post-COVID (15–60 days). Because infection and symptoms may vary across individuals, −14–0 days were used as an approximate pre-diagnosis window rather than a precise incubation interval.

**Results:**

Among 417 metabolomic features, 10 showed significant changes between baseline and PIP. Two metabolites, α-guanidinoglutaric acid and N-acetylneuraminic acid, were identified through library matching, while the remainder were characterized by mass and retention time. Temporal analysis revealed both transient metabolic shifts, which returned to baseline, and persistent changes, which remained altered post-COVID.

**Conclusions:**

These findings suggest that early metabolic changes before COVID-19 diagnosis may be detected in routine serum samples, offering opportunities to develop predictive models for early detection. Identifying these unique metabolomics fingerprints could improve personalized surveillance strategies and enhance understanding of COVID-19’s impact on hemodialysis patients.

## Introduction

Patients with kidney failure on hemodialysis (HD) are disproportionately impacted by coronavirus disease 2019 (COVID-19), a potentially life-threatening disease caused by the severe acute respiratory syndrome coronavirus 2 (SARS-CoV-2) [1]. In 2020, the mortality rate among HD patients with COVID-19 was 26% [2]. Later in the pandemic, a study conducted during the Omicron variant wave reported an all-cause mortality rate of 5.2% in HD patients between December 2022 and February 2023, with 2.7% caused by COVID-19 alone [3]. This population showed a high risk of death from COVID-19, which was associated with older age and prevalent comorbidities, such as diabetes and cardiovascular diseases [4]. COVID-19 screening has become essential to identify infected individuals. However, testing is not always available, and reverse transcriptase polymerase chain reaction (RT-PCR) test results are not immediate. Timely identification of infected individuals is critical to reduce the risk of severe outcomes, slow SARS-CoV-2 spread, support clinical decision-making, and improve patient care from an early stage of COVID-19.

The metabolome reflects the genome, transcriptome, and proteome, and provides a dynamic snapshot of an individual’s current metabolomic phenotype [5]. Using advanced techniques, such as liquid chromatography/mass spectrometry (LC/MS), it is possible to analyze a wide range of metabolites in a biological sample in an untargeted manner [5,6]. In this context, untargeted metabolomic profiling is intended as a discovery approach to identify candidate markers of presymptomatic and early disease, rather than as a stand-alone diagnostic tool. Such markers may subsequently enable the development of targeted, clinically deployable assays suitable for routine laboratory testing or point-of-care applications. Consistent with this rationale, LC/MS has revealed alterations in metabolomic profiles following COVID-19 infection with high potential for biomarker discovery [7]. Important pathways for cellular function, such as the tricarboxylic acid cycle, amino acid and lipid metabolism, are impaired in patients with COVID-19 [8,9]. Additionally, longitudinal trends indicate distinct metabolic profiles over the disease course. These profiles were related to the disease outcome and led to the creation of a panel of biomarkers that can potentially predict disease severity in the general population [10].

To the best of our knowledge, metabolomic changes before, during, and after COVID-19 in the HD population have not yet been studied. Furthermore, metabolomic signatures in the HD population remain scarce and warrant attention, as chronic kidney disease (CKD) itself induces metabolic alterations, independent of COVID-19. Therefore, it is conceivable that HD patients with COVID-19 will have metabolomic profiles that differ from the general population [11,12]. To address this issue, we performed untargeted metabolomics in leftover specimens of routinely collected serum samples of HD patients with COVID-19 between 60 days before and 60 days after infection. We aimed to (a) identify early alterations in the metabolome of HD patients in the two weeks before COVID-19 diagnosis and (b) evaluate their longitudinal metabolomic profile.

## Materials and methods

### Study design

This prospective, multi-center, observational study was conducted at four dialysis centers in New York City, NY, USA, from July 2021 to December 2022. The recruitment period for this study was from July 1 to December 31, 2021. All study procedures were approved by the Western Institutional Review Board (#1311160) and performed in accordance with the principles of the Declaration of Helsinki. Signed written informed consent was obtained from all participants undergoing in-center high flux HD. Inclusion criteria were age of 18 years or older, CKD treated with in-center HD, ability to give informed consent, and being on HD for at least 3 months. Exclusion criteria were cognitive impairment, life expectancy less than 12 months (e.g., metastatic cancer), and inability to communicate in English or Spanish.

### Patient selection and grouping

COVID-19 was diagnosed by a positive SARS-CoV-2 RT-PCR test of nasopharyngeal swabs. The onset of symptoms is usually patient-specific because the physiological reaction to an infection is variable. Therefore, the onset of symptoms relative to diagnosis is inevitably accompanied by some degree of uncertainty. As a result, COVID-19 phases were classified as baseline (days −60 to −15 before COVID-19 diagnosis), putative incubation period (PIP; −14 to day 0), acute (days 1–14), and post-COVID (days 15–60). Demographic, mortality, and hospitalization data were obtained from the patient’s electronic medical record.

### Patient samples

Serum samples (SST^TM^, Becton Dickinson, NJ, USA) collected as part of routine clinical care were sent from the dialysis clinics to Spectra Laboratories (NJ, USA). Upon completion of routine diagnostics, Spectra Laboratories stored leftover serum samples of study participants at −20°C until weekly shipment to the Renal Research Institute laboratory, where they were stored at −80°C until metabolomics analysis.

### Metabolite extraction from patient samples

Serum samples were thawed on ice, and the extraction order was randomized to minimize systematic biases. Stable isotope-labeled internal standards from the QReSS kit (Cambridge Isotope Laboratories, MA, USA, catalog #MSK-QRESS-KIT) were reconstituted in 50% methanol per manufacturer’s instructions. Metabolite extraction was performed using an automated liquid handler (Biomek 4000, Beckman Coulter, IN, USA). Briefly, 25 µL of freshly prepared, 10x diluted internal standard solution was added to 50 µL of serum, followed by 150 µL of ice-cold methanol. Samples were vortexed and centrifuged at 4300 rpm for 15 minutes at 4°C. Fifty µL of the supernatant were transferred to a chilled 96-well plate, dried at 20°C using a centrifugal vacuum evaporator (Labconco, MO, USA), and stored at −80°C until analysis.

### Liquid chromatography/mass spectrometry analysis

On the day of analysis, dried extracts were reconstituted in 50 µL of 50% mobile phase A (0.01% formic acid and 1 mM ammonium formate in ultrapure water) and 50% mobile phase B (0.01% formic acid and 1 mM ammonium formate in methanol) and maintained at 4°C in the multisampler. Five µL were injected in randomized order into an Agilent 1290 Infinity II liquid-chromatography system. Separation was performed on a reverse-phase C18 column (Luna C18, 4.6 × 150 mm, 3 µm, Phenomenex, CA, USA) with a guard column (SecurityGuard^TM^, Phenomenex, CA, USA) at a flow rate of 0.75 mL/min using a linear gradient: 1% B from 0.5 to 7 min, 40% B from 7 to 8.5 min, 90% B from 8.5 to 12 min, 100% B from 12 to 12.5 min, followed by a 3 min re-equilibration at initial conditions.

Untargeted acquisition was performed on a Quadrupole-Time-of-Flight mass spectrometer (QToF 6546, Agilent Technologies, CA, USA) using electrospray ionization in positive mode. The source parameters were as follows: gas temperature 350°C, gas flow 12 L/min, sheath gas temperature 400°C, sheath gas flow 12 L/min, nebulizer 35 psig, fragmentor 125 V, skimmer 65 V, nozzle voltage 500 V, VCap 4000 V, and *m/z* range of 50–1100. MS2 spectra were acquired in iterative mode at 3 spectra per second using collision energies of 10, 20, and 40 eV.

### Quality control to monitor analytical variance

Quality control (QC) consisted of pooled samples prepared after metabolite extraction for each batch. Ten QC injections were used to condition the column, followed by one QC injection after every five samples to monitor signal stability. To validate the signal response, diluted QC samples were analyzed at the end of each batch. Blank injections (50% mobile phase A, 50% mobile phase B) and process blanks (blanks that went through the metabolite extraction procedure) were used to identify and remove background ions and contaminants. Instrument maintenance and calibration were performed before each batch according to standard operating procedures. Batch performance was evaluated by overlap of QC total ion chromatograms and by calculating coefficients of variation (CV) of internal standard signals, with acceptance defined as CV ≤ 10%.

### Data mining and feature extraction

Feature extraction and retention time alignment for each batch were performed using Profinder v10.0 software (Agilent Technologies, CA, USA), followed by data cleaning with Mass Profiler Professional v15.1 (Agilent Technologies, CA, USA). Contaminants and background ions were removed, and features not present in 100% of QC injections were excluded. Features with CV ≥ 20% that did show appropriate dilution response in diluted QC were further filtered. Feature lists from four batches were integrated by matching *m/z* (± 5 ppm) and retention time (± 0.2 min). Subsequent analyses were performed using R statistical software (R Foundation for Statistical Computing, Vienna, Austria; R Core Team 2021). Features with >50% missing values were removed from the dataset. The intensities of the remaining features were log2-transformed to mitigate data distribution skewness. For the imputation of the remaining missing values, the random forest-based MissForest algorithm [13] was used (R package missForest; Stekhoven, 2022).

### Batch correction

Principal component analysis (PCA) was used to assess batch effects. Two-dimensional principal component (PC) plots revealed batch effects in the data, and ComBat [14] was applied to correct them (R package sva; Leek et al, 2022).

### Metabolite identification

Metabolite identifications were achieved by (with decreasing priority) (1) matching accurate mass, retention time, and MS2 fragmentation pattern with a library built in-house, or (2) matching accurate mass and retention time with the same library built in-house, or (3) matching accurate mass and fragmentation pattern with MassHunter METLIN (Agilent Technologies, CA, USA). The in-house library was created using reference standards (MetaSci, ON, Canada). It comprises data from 703 metabolites acquired using the same instrument and LC/MS method of study samples.

### Statistical inference using linear mixed-effects model (LMEM)

For each feature, we tested whether intensity differed between baseline (days −60 to −15) and PIP (days −14–0) using a linear mixed-effects model. Intensities were analyzed on a log2 scale; model estimates can be interpreted as fold changes relative to baseline (e.g., 0 = no change; 1 = 2-fold increase). A detailed description of the analysis is provided in **S1 File**.

### Trajectory analysis using semi-parametric linear mixed-effects model (SLMEM)

To capture non-linear temporal patterns, we modeled log2 intensity across all time points using a semi-parametric mixed-effects model with a cubic spline for time and a patient-specific random intercept. Fitted trajectories and 95% confidence intervals are shown. A detailed description of the method is provided in **S2 File**.

## Results

### Study population and samples

**Fig 1:**
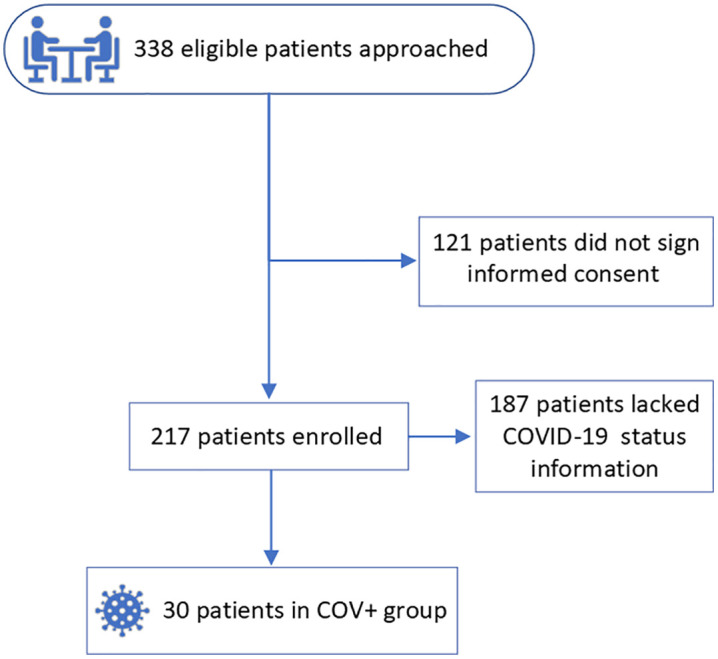
Study flowchart. COV+ , hemodialysis patients with nasopharyngeal swab tested positive for SARS-CoV-2 by reverse transcriptase polymerase chain reaction (RT-PCR).

**Fig 2:**
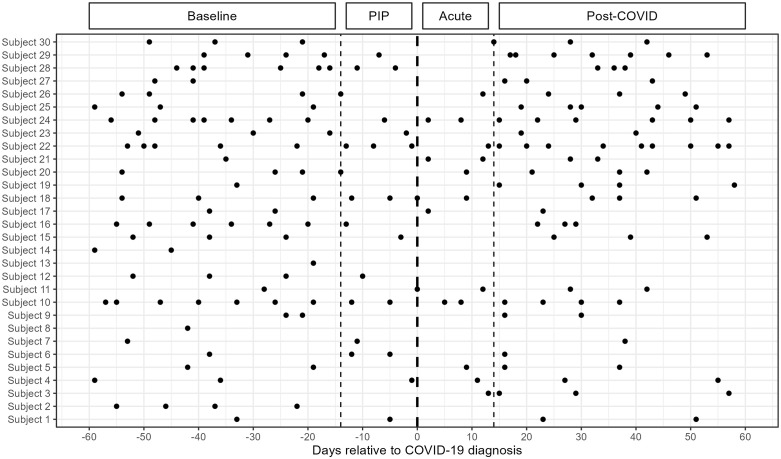
Temporal distribution of longitudinal metabolomics samples. The dashed vertical lines show borders between the four COVID-19 phases: baseline (days −60 to −15), putative incubation period (PIP; −14 to day 0), acute (days 1 to 14), and post-COVID (days 15 to 60). Each point represents a sample from the respective COV+ study subject indicated on the y-axis. A total of 30 hemodialysis patients with confirmed COVID-19 were investigated, providing 201 serum samples.

We screened 338 HD patients, and 217 of them consented to the study. We evaluated the serum metabolomics profile of 30 patients who tested positive for COVID-19 during the study period (**Fig 1**). Their average age was 59.2 ± 13.3 years; 57% were male, 63% were Black, 20% were Hispanic, 30% had diabetes, 57% had hypertension, 3% had congestive heart failure, and dialysis vintage was 4.4 ± 4.7 years. COVID-19 led to hospitalization in six patients and resulted in mortality in two patients. A total of 201 samples, collected within 60 days before and after the COVID-19 diagnosis date, were analyzed. Of these samples, 83 were collected during baseline, 24 during the PIP, 16 during acute, and 78 post-COVID (**Fig 2**).

### Batch effect correction

**Fig 3:**
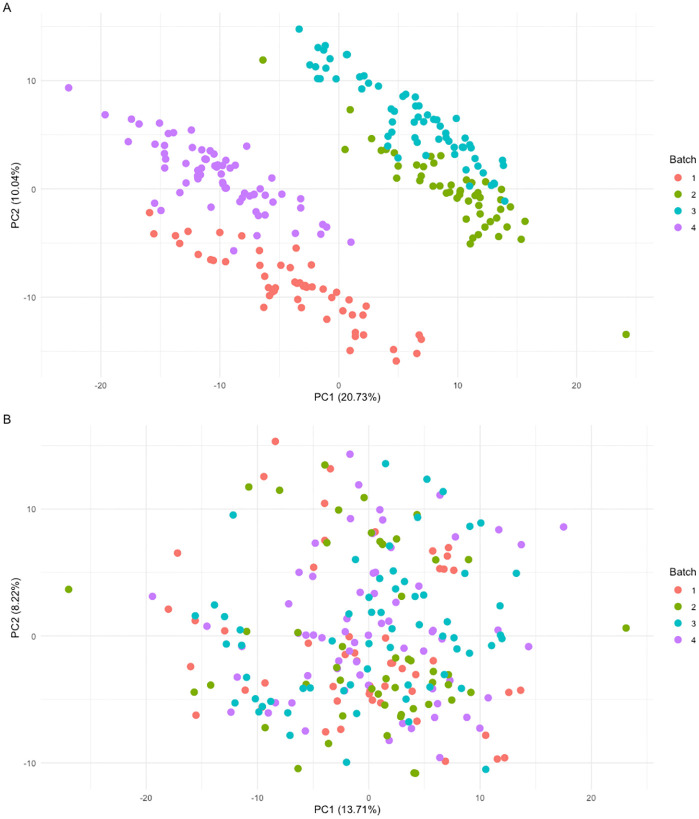
Two-dimensional principal component (PC) plots of metabolic features before (A) and after (B) ComBat batch correction. PC1 and PC2 represent the first and second principal components, respectively, which reflect the top two orthogonal directions that capture the most variation in the data. Red, green, blue, and purple dots represent samples from batches 1, 2, 3, and 4, respectively.

The final dataset included 417 metabolic features with corresponding peak areas for each sample. Since samples were analyzed in four different batches, we observed batch-related effects, as shown in the two-dimensional PC plot (**Fig 3A**). After applying the empirical Bayes ComBat normalization method, batch effects were minimized while biological variation was maintained. The two-dimensional PC plot based on the normalized data no longer showed clusters in batches (**Fig 3B**).

### Linear mixed-effects model (LMEM) analysis of baseline (−60 to −15 days) and PIP (−14–0 days)

**Fig 4:**
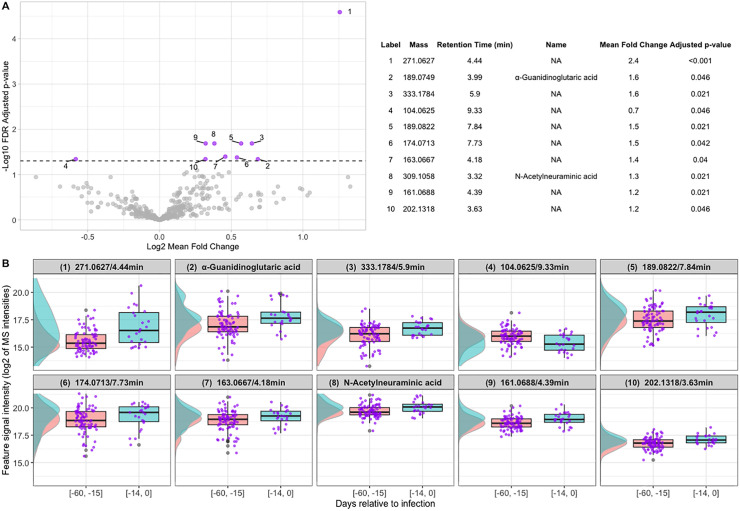
Metabolic feature levels at baseline and the putative incubation period (PIP). (A) The Volcano plot presents the log2 mean fold change from the baseline level (days −60 to −15) to the level during PIP (−14 to day 0) and the log10 FDR-adjusted p-value of each metabolic feature. The dashed line represents the FDR-adjusted p-value cutoff of 0.05. Features with a mean fold change of ≥ 0.7 are highlighted. (B) The boxplots and density functions show the distribution of each significant metabolic feature in feature signal intensity (log2) compared to days relative to infection at the baseline [−60, −15] and PIP [−14, 0]. Features are labeled with names if identified. In the absence of annotated names, features are designated by their accurate mass and retention time, for example, Feature 1 (271.0627/4.44 min).

We investigated metabolomic alterations by fitting the LMEM (**S1 File**) for each feature and testing the hypothesis that *β*_1_ = 0. The LMEM analysis identified 10 significant metabolic features with Benjamini and Hochberg false discovery rate (FDR) [15]-adjusted p-values < 0.05. Of the 10 features, both increased and decreased features were retained. Two features were identified as α-guanidinoglutaric acid (average 1.6-fold increase; FDR-adjusted p-value = 0.046) and N-acetylneuraminic acid (average 1.3-fold increase; FDR-adjusted p-value = 0.021) (**S1 Fig**), both with higher levels at the PIP compared to baseline (**Fig 4A and 4B**). MS2 spectra of the currently unidentified features are provided in **S2 Fig**. The distributions of the 10 significant features in both disease phases are shown in **Fig 4B**. The density curves help visualize the shapes of distributions of feature samples, where the peak of the curve indicates the mode and the width indicates the variation. For the full list of 417 metabolic features and their associated LMEM results, see **S1 Table**.

### Metabolic changes from baseline to post-COVID

**Fig 5:**
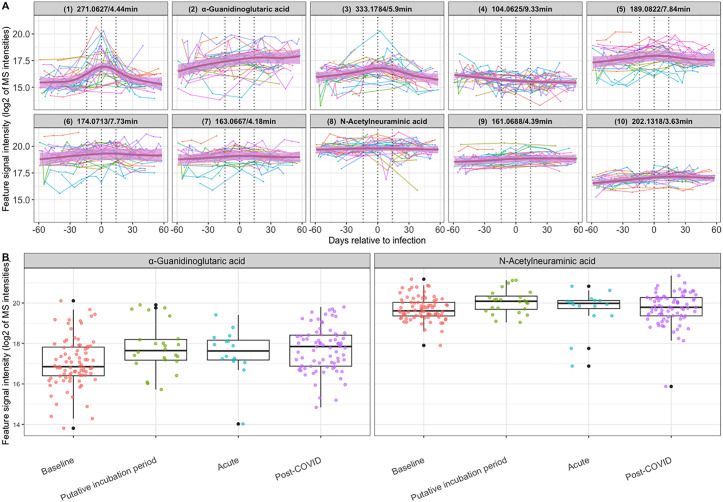
Profiles of significant metabolic features over time. **(A)** The vertical dashed lines indicate the separation of the time windows baseline (days −60 to −15), the putative incubation period (PIP) (−14 to day 0), acute (days 1 to 14), and post-COVID (days 15 to 60). Features are shown as log2-transformed peak area signal intensities. Non-identified features are labeled as accurate mass/retention time (min). The pink line and pink region in each plot are the Semi-parametric Linear Mixed Effect Model (SLMEM) curve and its 95% confidence region, respectively, reflecting the trend of the feature intensities. The details of SLMEM are provided in **S2 File****. (B)** The box-and-whisker plots show the distributions of α-guanidinoglutaric acid and N-acetylneuraminic acid at baseline, PIP, acute, and post-COVID.

We used the identified 10 features with alterations in the PIP to evaluate their metabolic fluctuations from baseline to ost-COVID. **Fig 5A** shows longitudinal levels of these metabolic features in each patient, as well as the semi-parametric linear mixed effect model (SLMEM) [16] curves that estimate their trajectories over time [17]. Interestingly, features 1 and 3 exhibited a pattern of rising levels during the PIP, returning to baseline in the post-COVID phase (**Fig 5A**). Feature 4 showed a downward trend, which did not return to baseline levels post-COVID (**Fig 5A**). For the two annotated metabolites, N-acetylneuraminic acid increased in the PIP and returned to baseline levels afterward, and α-guanidinoglutaric acid levels remained high throughout the disease phases (**Fig 5A** an**d 5B**).

We also examined metabolic changes from 60 days before diagnosis to day 0 in 22 serum samples from six patients who were hospitalized because of COVID-19 and in 69 samples from 18 patients who were not hospitalized (**S3 File**). A total of 66 metabolic features differed significantly between the two groups after FDR adjustment (p < 0.05), including piperine, L-phenylalanine, N-acetylneuraminic acid, N-alpha-acetyl-L-arginine, and 2-pyrrolidone-5-carboxylic acid (**S2 Table**). These results are exploratory and, due to the small sample size, provide preliminary evidence for a potential pre-diagnostic signature of disease severity.

## Discussion

This is the first study in maintenance HD patients infected with SARS-CoV-2 that explored the temporal evolution of serum metabolic features from 60 days before to 60 days after COVID-19 diagnosis. We discovered distinct dynamics of α-guanidinoglutaric acid, N-acetylneuraminic acid, and 8 unidentified metabolic features during this time window. Although the observed fold changes were modest, such shifts are consistent with early disease states and tightly regulated biological systems, where reproducible but subtle metabolic perturbations often precede overt clinical manifestations. These findings may provide insight into early biological changes associated with infection rather than serving as standalone diagnostic markers.

The collection of metabolites in an organism – commonly accessed by LC/MS – provides a snapshot of the current metabolomic phenotype. Increased availability of LC/MS in medical laboratories facilitates the application of metabolomics-based approaches for diagnosis, prognosis, early detection, and development of therapeutic strategies [18]. Importantly, the relevance of the findings herein lies in the early emergence of the biological signal rather than the analytical speed of untargeted LC/MS workflows, which remain resource-intensive and are not intended to replace RT-PCR. Instead, this discovery-phase approach is intended to inform the development of simplified, targeted biomarker panels that could be implemented within existing centralized dialysis laboratory workflows, using streamlined, automated assays with substantially shorter turnaround times.

The present study was conducted in a maintenance dialysis population. A comparison of the results with published metabolomic profiles from the general population suggests both shared and potentially unique features of the host response to COVID-19 infection. Several studies in the general population have explored metabolomics alterations following COVID-19. An extensive overview is given by Bourgin et al. 2023 [7]. Some of the metabolic shifts associated with COVID-19 reflect dyslipidemia, proteolysis, and kidney and liver dysfunction [8,19]. All but one study [20] evaluated biological samples collected in patients with active COVID-19, while subjects without COVID-19 served as controls [8,9,21–25]. Consistently, lipid levels were altered during COVID-19 infection. For instance, free fatty acids, arachidonic acid, and oleic acid were correlated with disease severity in a study conducted early in the pandemic [22]. A longitudinal study profiling plasma samples after COVID-19 diagnosis through recovery identified a panel of 22 metabolites that were predictive of COVID-19-related admissions to an intensive care unit. Different lipid classes (i.e., lysophosphatidylcholines and phosphatidylethanolamines) and polar metabolites (i.e., kynurenate and 1-methyladenosine) significantly contributed to this metabolic panel [10].

Chatelaine et al. (2023) [20] conducted the first longitudinal study of COVID-19-related metabolic alterations in the general population before, during, and after infection. Metabolites of the sphingolipid, phospholipid, and amino acid metabolism were among the ones that did not restore to baseline levels within 28 days of diagnosis. Additionally, the authors found a significant association of N-acetylneuraminic acid with COVID-19 severity before and during infection. We observed increased levels of N-acetylneuraminic acid in the PIP in the HD population. N-acetylneuraminic acid is member of a class of sugars called sialic acids, which are naturally found attached to glycans on all cell types [26]. This overlap between the general and HD population suggests that certain metabolic responses to SARS-CoV-2 infection are conserved, while their temporal dynamics and magnitude may be modulated by dialysis and the uremic milieu. Nguyen et al. (2022) [27] found that host sialylated glycans are a target for SARS-CoV-2 binding, which may contribute to viral entry. A recent study reported acetyl-4-O-acetylneuraminic acid, another sialic acid, as a potential biomarker of COVID-19 diagnosis and prognosis [28], corroborating the importance of this class of compounds during COVID-19.

In humans, information about α-guanidinoglutaric acid – the second annotated metabolite presented herein – is very limited. Nonetheless, in CKD patients, guanidino compounds such as guanidinosuccinic acid, γ-guanidinobutyric acid, and β-guanidinopropionic acid are classified as water-soluble uremic toxins by the EuTox database (https://www.uremic-toxins.org/) [29–31]. In fact, increased levels of guanidino compounds in the brain of uremic patients were previously reported and may be linked to neurological complications associated with CKD [32], given that these compounds have limited brain-to-blood efflux [33]. It was shown that intraventricular administration of α-guanidinoglutaric acid in rats induced epileptic seizures [34]. However, neurological outcomes were not assessed in our study cohort. Sustained elevated levels of α-guanidinoglutaric acid from the PIP to the post-COVID phase presented herein warrant further evaluation. In contrast to N-acetylneuraminic acid, the prominence of guanidino compounds in CKD suggests that this metabolic signal may represent a kidney disease-specific response to SARS-CoV-2 infection rather than a universal COVID-19 marker. Future comparative work in larger cohorts with matched non-HD controls will be required to delineate universal COVID-19 signatures from those specific to the dialysis population.

In addition to α-guanidinoglutaric acid and N-acetylneuraminic acid, we detected 8 metabolic features with alterations during the PIP phase. Incomplete metabolite identification restricts mechanistic interpretation and precludes immediate clinical application. Importantly, these findings should be viewed as hypothesis-generating. Future work will focus on targeted validation in a larger cohort. Although we could not annotate these compounds, this fingerprint may aid in metabolic profiling. Similar to the observed longitudinal trends for the 10 significantly changed features, others have assessed metabolite levels during and after infection in the general population and found a tendency for up- or downregulation toward levels observed in non-infected individuals, although a few metabolic changes persisted after recovery [10,35]. Factors such as dialysis vintage, comorbidities, and medication use may influence metabolic profiles. The results presented herein serve as a starting point for larger studies in which such potential confounders can be explicitly incorporated as covariates.

In conclusion, we discovered that α-guanidinoglutaric acid, N-acetylneuraminic acid, and 8 currently unidentified metabolomic features change in response to SARS-CoV-2 infection. These findings may shed light on the biology of COVID-19 in HD patients. Additional studies are warranted to explore the associations between these metabolomic changes and patient characteristics and outcomes.

## Supporting information

S1 Fig Spectral match results for annotated metabolites. Top panel: MS2 spectra of a quality control sample. Middle spectra panel: comparison between MS2 spectra of quality control sample and the standard in the library (“mirror plot”). Bottom spectra panel: MS2 spectra of the standard in the library. (a) N-acetylneuraminic acid; (b) α-Guanidinoglutaric acid.
(PDF)

S2 Fig MS2 spectra of the currently unidentified features. Note that MS2 spectra for feature 1 cannot be reliably extracted.
(PDF)

S1 Table Linear mixed-effects model results for metabolic features. (XLSX)

S2 Table Metabolic features in hospitalized vs non-hospitalized patients. (XLSX)

S1 File Detailed description of linear mixed-effects model. (PDF)

S2 File Detailed description of semi-parametric linear mixed effect model. (PDF)

S3 File Detailed description of Welch’s t-test [36]. (PDF)
