# Supplementary material for: COVID-19 in hemodialysis patients: New insights into metabolomic profile dynamics from 60 days pre- to 60 days post-diagnosis
Source: PLoS One. 2026 Apr 17;21(4):e0346687. doi: 10.1371/journal.pone.0346687 (PMC13089734; doi:10.1371/journal.pone.0346687)
Supplement: S1 Fig — Top panel: MS2 spectra of a quality control sample. Middle spectra panel: comparison between MS2 spectra of quality control sample and the standard in the library (“mirror plot”). Bottom spectra panel: MS2 spectra of the standard in the library. (a) N-acetylneuraminic acid; (b) α-Guanidinoglutaric acid. (PDF) [file pone.0346687.s001.pdf]

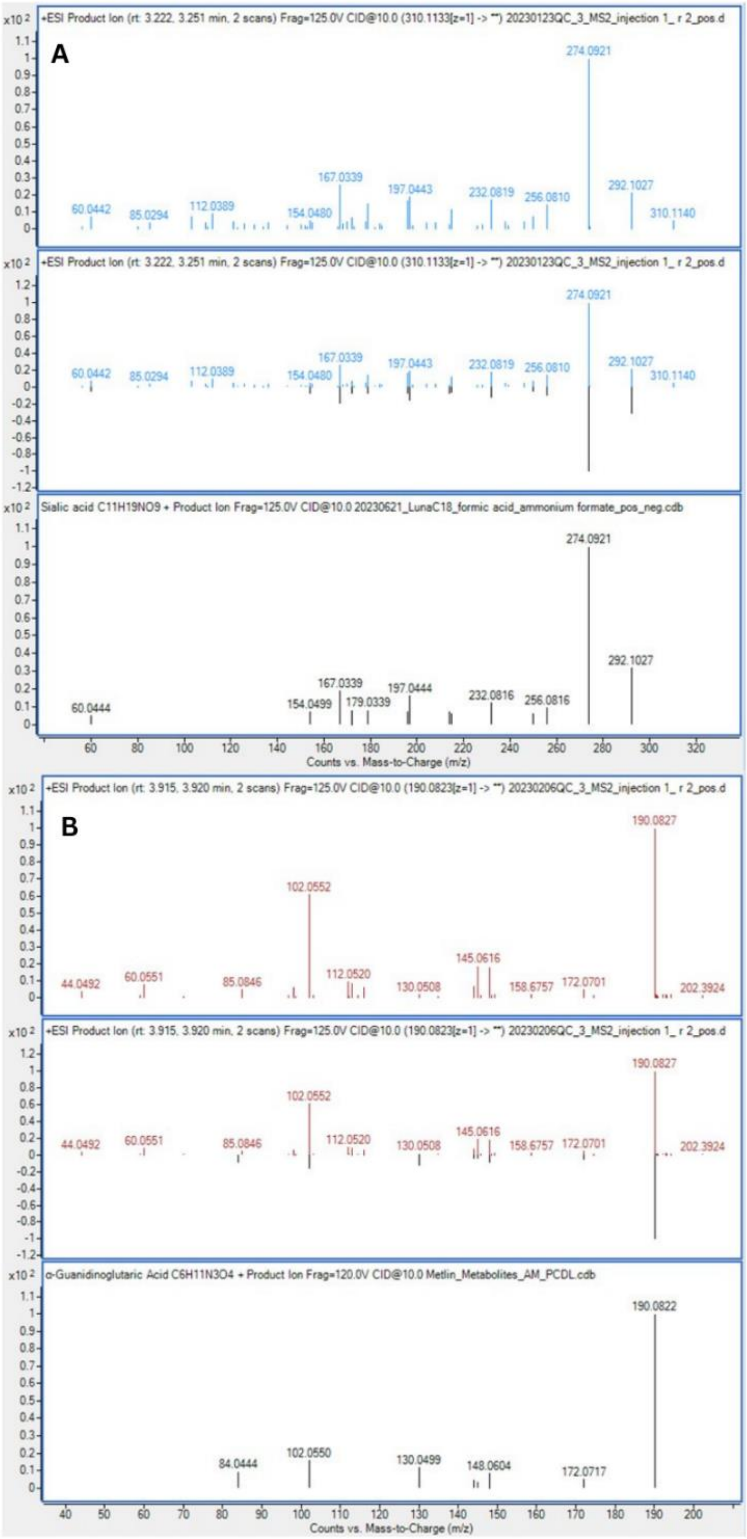

S1 Figure. Spectral match results for annotated metabolites. Top panel: MS2 spectra of a quality control sample. Middle spectra panel: comparison between MS2 spectra of quality control sample and the standard in the library (“mirror plot”). Bottom spectra panel: MS2 spectra of the standard in the library. (a) N-acetylneuraminic acid; (b) α-Guanidinoglutaric acid.
