# Supplementary material for: COVID-19 in hemodialysis patients: New insights into metabolomic profile dynamics from 60 days pre- to 60 days post-diagnosis
Source: PLoS One. 2026 Apr 17;21(4):e0346687. doi: 10.1371/journal.pone.0346687 (PMC13089734; doi:10.1371/journal.pone.0346687)
Supplement: S2 Fig — Note that MS2 spectra for feature 1 cannot be reliably extracted. (PDF) [file pone.0346687.s002.pdf]

Feature 3

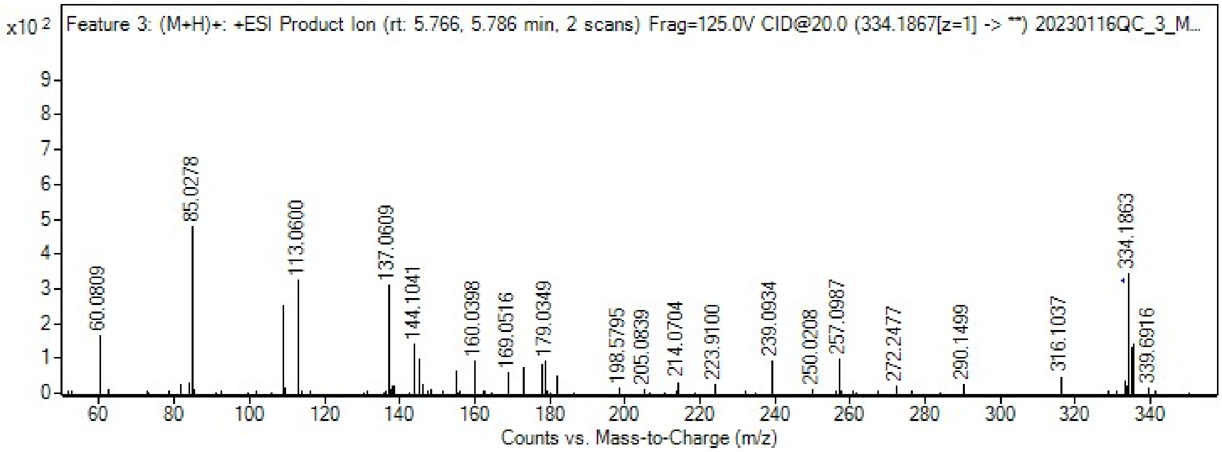

Feature 4

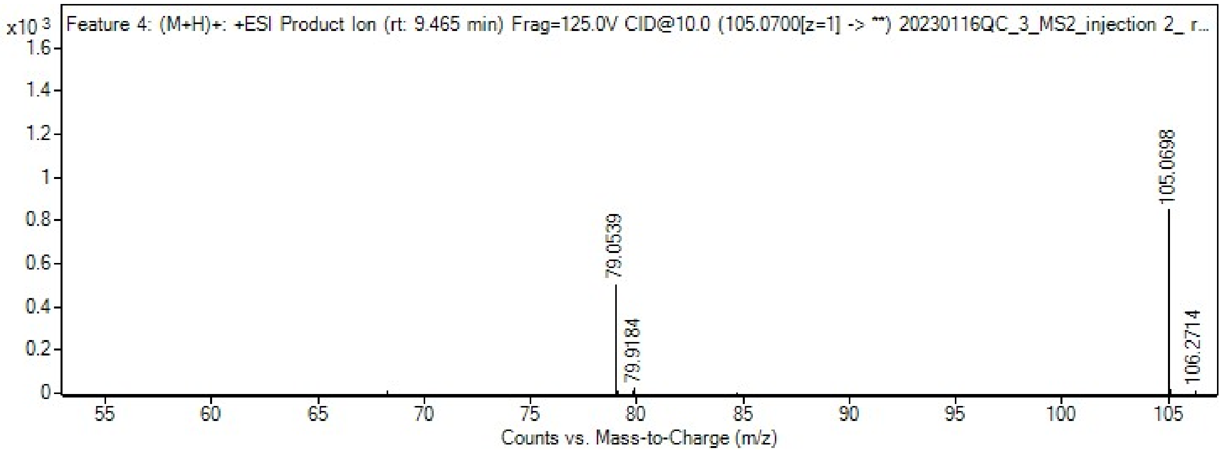

Feature 5

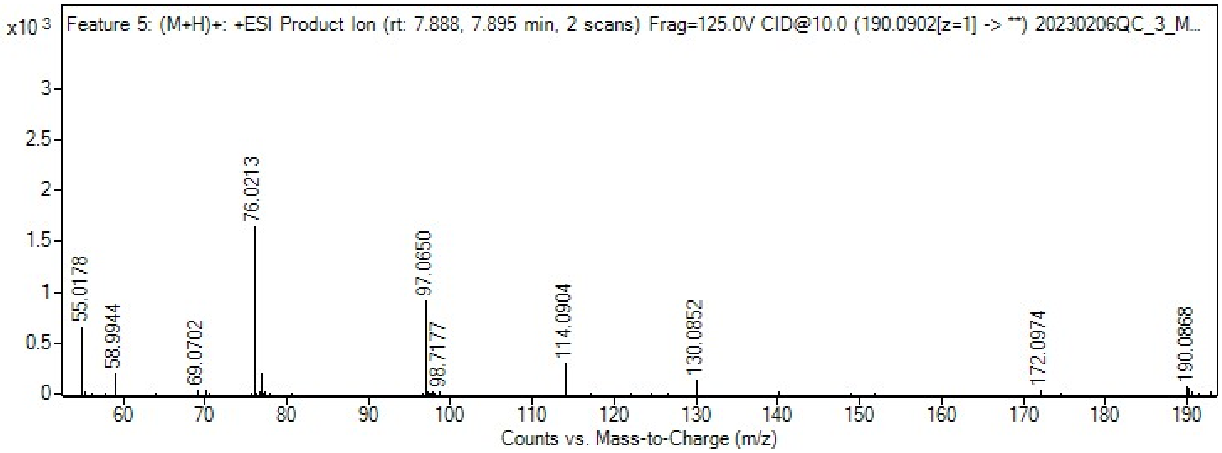

Feature 6

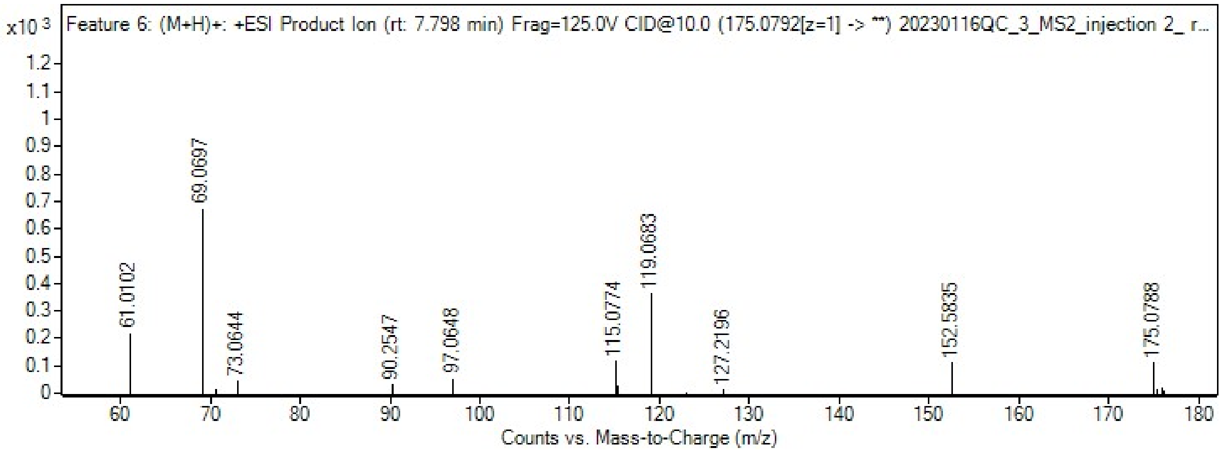

Feature 7

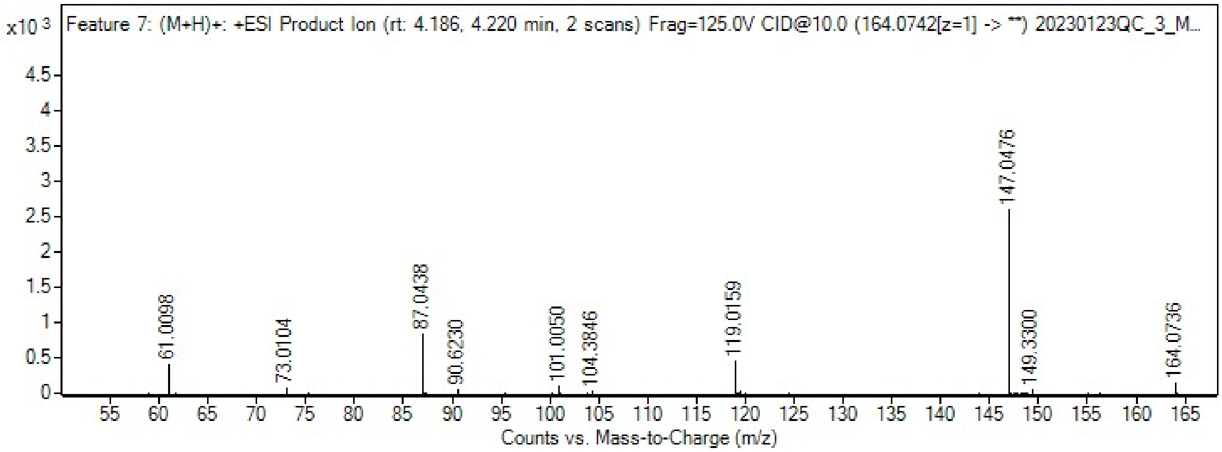

Feature 9

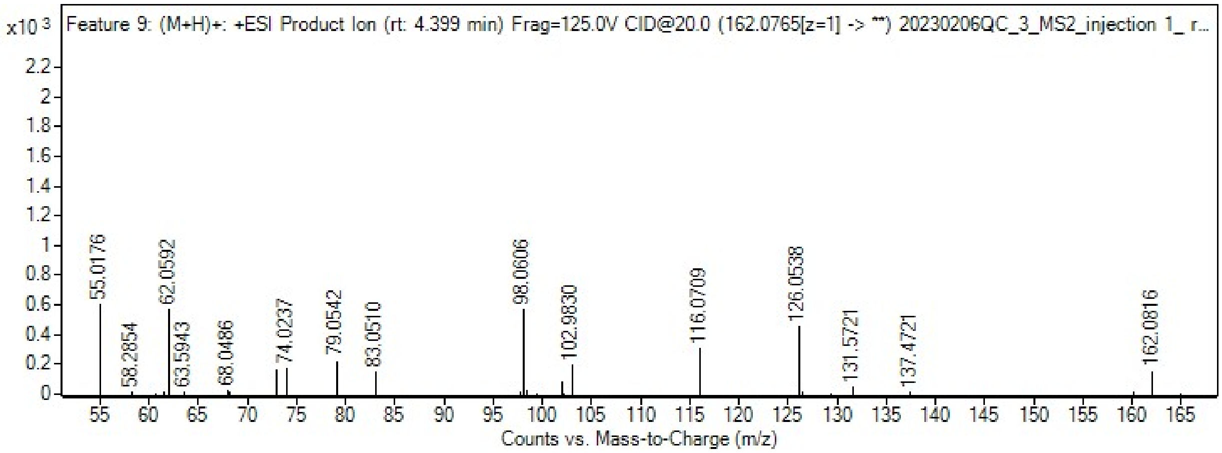

Feature 10

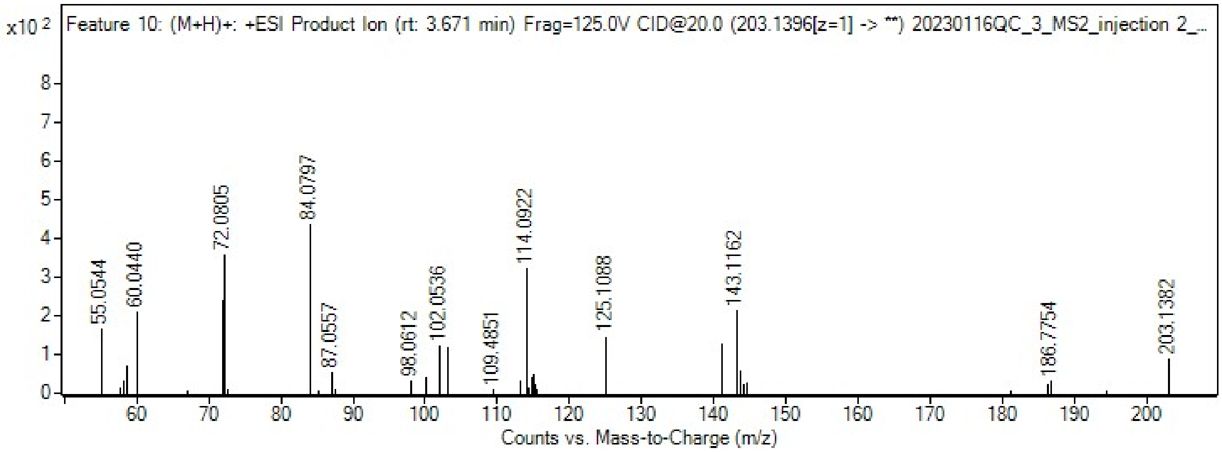

S2 Figure. MS2 spectra of the currently unidentified features. Note that MS2 spectra for feature 1 cannot be reliably extracted.
