# Supplementary material for: COVID-19 in hemodialysis patients: New insights into metabolomic profile dynamics from 60 days pre- to 60 days post-diagnosis
Source: PLoS One. 2026 Apr 17;21(4):e0346687. doi: 10.1371/journal.pone.0346687 (PMC13089734; doi:10.1371/journal.pone.0346687)
Supplement: S1 File — (PDF) [file pone.0346687.s005.pdf]

S1 File. Detailed description of Linear Mixed-Effects Model

For each feature, we fit the following linear mixed-effects model using COV+ patients' samples in the putative incubation period and baseline phase:

$$Y_{ij} = \beta_0 + \beta_1 \times \mathrm{I}_{\{\text{Putative incubation period}\}} + b_i + e_{ij}$$

where $Y_{i,j}$ is the log2 transformed observed intensity level of the feature in the j-th sample from the i-th patient, $\mathrm{I}_{\{\text{Putative incubation period}\}}$ is the indicator function taking value 1 if the current sample belongs to the putative incubation period and 0 otherwise, β_0 is the feature average intensity level for the COV+ population at the baseline phase, β_1 is the difference between the feature average intensity of the putative incubation period and baseline phase, e_{ij} is the random error following iid normal distribution with mean 0 and variance σ^2. The random effect for each patient i, b_i, is introduced to account for inter-patient variability. The random effect b_i is assumed to be independent and identically distributed (iid) from the normal distribution with mean 0 and variance σ_b^2. The random effects and random errors are mutually independent.

The parameter β_1 represents the average difference between baseline and PIP and 2^{β_1} corresponds to the average ratio (fold change) between the two time periods. We tested the hypothesis of $\beta_1 = 0$ to identify features with significant intensity changes in the PIP compared to the baseline phase. The Benjamini and Hochberg false discovery rate (FDR) correction [15] is used to control the FDR. The same methods are used to compute the p-values of β_1 for all features and the features with an FDR-adjusted p-value < 0.05 were retained for further analysis.
