# Supplementary material for: COVID-19 in hemodialysis patients: New insights into metabolomic profile dynamics from 60 days pre- to 60 days post-diagnosis
Source: PLoS One. 2026 Apr 17;21(4):e0346687. doi: 10.1371/journal.pone.0346687 (PMC13089734; doi:10.1371/journal.pone.0346687)
Supplement: S2 File — (PDF) [file pone.0346687.s006.pdf]

S2 Text. Detailed description of Semi-parametric Linear Mixed Effect Model.

To capture the non-linear patterns in the sample and show the trajectory of the samples for each feature, we fit the following semi-parametric linear mixed-effect model using COV+ patients' samples across all time points:

$$Y_{ij} = f(t_{ij}) + b_i + \epsilon_{ij},$$

where Y_{ij} is the log2 transformed observed intensity level of the feature in the j-th sample from the i-th patient, $f \in W_2^2[0,1]$ is a non-parametric function in the Sobolev space on [0, 1],

$$W_2^2[0,1] = \left\{f\colon f,\, f',\, f^{(2)} are\ absolutely\ continous,\ \int_0^1 \left(f^{(2)}\right)^2 dx < \infty\right\},$$

where t_{ij} is the observational time point scales into the interval [0,1], b_i is the random effect for the i-th patient, $\epsilon_{ij}$is the random error following iid normal distribution with mean 0 and variance σ^2. The random effects b_i are assumed to be independent and identically distributed (iid) from the normal distribution with mean 0 and variance σ_b^2. The random effects and random errors are mutually independent. The fitted values and 95% confidence intervals of the model are plotted as smooth curves and shaded bands for each feature to reflect the trend of samples over time.
