# Supplementary material for: COVID-19 in hemodialysis patients: New insights into metabolomic profile dynamics from 60 days pre- to 60 days post-diagnosis
Source: PLoS One. 2026 Apr 17;21(4):e0346687. doi: 10.1371/journal.pone.0346687 (PMC13089734; doi:10.1371/journal.pone.0346687)
Supplement: S3 File — (PDF) [file pone.0346687.s007.pdf]

S3 File. Detailed description of Welch's t-test.

Welch's t-test, also named the unequal variances t-test, is a statistical method used to test the null hypothesis that two independent populations have equal means, without assuming that the populations have equal variances [36]. The hypotheses for Welch's t-test are that the population means of the two groups are equal under the null hypothesis (H_0: $\mu_1 = \mu_2$), and the alternative hypothesis for a two-tailed test states that the population means are not equal (H_A: $\mu_1 \neq \mu_2$).

The test statistic for Welch's t-test is:

$$t = \frac{\overline{X}_1 - \overline{X}_2}{\sqrt{\frac{s_1^2}{n_1} + \frac{s_2^2}{n_2}}},$$

where $\overline{X}_1$ and $\overline{X}_2$ are the sample means of groups 1 and 2, s_1^2 and s_2^2 are the sample variances of groups 1 and 2, $n_1$and n_2 are the sample sizes of the groups 1 and 2.

The test statistic t follows t-distribution with degrees of freedom v , and v is approximated using the Welch-Satterthwaite equation [36]:

$$v \approx \frac{\left(\frac{s_1^2}{n_1} + \frac{s_2^2}{n_2}\right)^2}{\frac{\left(\frac{s_1^2}{n_1}\right)^2}{n_1 - 1} + \frac{\left(\frac{s_2^2}{n_2}\right)^2}{n_2 - 1}}.$$

For a two-tailed test, based on the calculated test statistic t , the p-value, which represents the probability of obtaining a test statistic as extreme as the observed one under the null hypothesis, will be computed as:

$$\text{p-value} = P_{H_0}(T \geq |t|) ,$$

where T follows t-distribution with degree of freedom v . The Benjamini and Hochberg false discovery rate (FDR) correction [15] is used to control the FDR, and the features with an FDR-adjusted p-value < 0.05 were retained for further analysis.
